# Paprika Pigments Attenuate Obesity-Induced Inflammation in 3T3-L1 Adipocytes

**DOI:** 10.1155/2013/763758

**Published:** 2013-04-11

**Authors:** Hayato Maeda, Shuuichi Saito, Nozomi Nakamura, Takashi Maoka

**Affiliations:** ^1^Faculty of Agriculture and Life Science, Hirosaki University, 3 Bunkyo-cho, Hirosaki, Aomori 036-8561, Japan; ^2^Research Institute for Production and Development, 15 Shimogamo-morimotocho, Sakyo-ku, Kyoto 606-0805, Japan

## Abstract

Obesity is related to various diseases, such as diabetes, hyperlipidemia, and hypertension. Adipocytokine, which is released from adipocyte cells, affects insulin resistance and blood lipid level disorders. Further, adipocytokine is related to chronic inflammation in obesity condition adipocyte cells. Paprika pigments (PPs) contain large amounts of capsanthin and capsorubin. These carotenoids affect the liver and improve lipid disorders of the blood. However, how these carotenoids affect adipocyte cells remains unknown. Present study examined the effects of PP on adipocytokine secretion, which is related to improvement of metabolic syndrome. In addition, suppressive effects of PP on chronic inflammation in adipocyte cells were analyzed using 3T3-L1 adipocyte cells and macrophage cell coculture experiments. PP promoted 3T3-L1 adipocyte cells differentiation upregulated adiponectin mRNA expression and secretion. Further, coculture of adipocyte and macrophage cells treated with PP showed suppressed interleukin-6 (IL-6), tumor necrosis factor-**α** (TNF-**α**), monocyte chemotactic protein-1 (MCP-1), and resistin mRNA expression, similarly to treatment with troglitazone, which is a PPAR**γ** ligand medicine. *Conclusion*. These results suggest that PP ameliorates chronic inflammation in adipocytes caused by obesity. PP adjusts adipocytokine secretion and might, therefore, affect antimetabolic syndrome diseases.

## 1. Introduction

Obesity has increased rapidly in recent years. It is currently regarded as a major risk factor for type 2 diabetes, hypertension, and dyslipidemia [1, 2]. The cluster of these three diseases is called metabolic syndrome, of which the incidence is a worldwide problem [3, 4]. Recent reports have described that obesity is characterized by low-grade chronic inflammation. Inflammation suggests a mechanism by which obesity engenders insulin resistance [5, 6]. Adipocytes are recognized as an important endocrine cell that secretes biologically active mediators called adipocytokines [4, 7]. They affect insulin sensitivity, glucose and lipid metabolism in muscle, liver, and adipose tissue and induce metabolic syndrome. TNF-*α* and resistin, adipocytokines, are known to be elevated in obesity. They play important roles in the development of insulin resistance and type 2 diabetes [8, 9]. MCP-1 induces the infiltration of macrophages into adipose tissue. Moreover, it enhances inflammation causing insulin resistance [10]. These adipocytokines, which are secreted actively from hypertrophy adipocytes in conditions of obesity, exacerbate blood glucose homeostasis in several organs. Therefore, it is conceivable that adjustment of adipocytokines secretion is important for suppressing glucose metabolic disorder.

**Figure 1:**
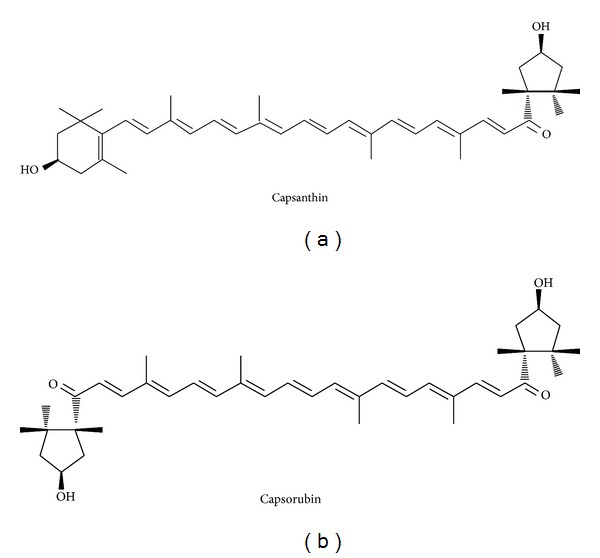
Structures of capsanthin and capsorubin in paprika pigments.

Paprika (*Capsicum annuum* L.) is widely used as a vegetable and food additive, commonly called sweet pepper. It contains red color pigments used as natural colorants in various foods. Capsanthin and capsorubin, which are major carotenoids present in paprika (Figure 1), are normally present in an acylated form with fatty acids in PP [11]. Capsanthin and capsorubin are both nonprovitamin A carotenoids with various activities such as antioxidation, anticancer, and anti-inflammatory effects [12, 13]. Recently, Aizawa and Inakuma reported that capsanthin in paprika has a plasma HDL-cholesterol raising effect accompanied by a significant increase in hepatic apoA5 and LCAT mRNA [14]. The high LDL-cholesterol level of blood is a risk factor in cardiovascular diseases, but HDL-cholesterol removes excess cholesterol from organs. Therefore, dietary intake of paprika pigments might improve plasma lipid profiles. Carotenoids are natural pigments contained in many vegetables and fruits. Dietary carotenoids are also known to reduce risk life-style diseases such as cardiovascular diseases, cancer, and diabetes [15–17]. For example, astaxanthin, which is founds in seafood and microalgae, ameliorates insulin resistance by promoting adiponectin secretion [18]. Fucoxanthin, which is found in brown seaweed, prevents obesity. Its related type 2 diabetes regulates adipocytokine expression [19, 20]. Various carotenoids in food are likely to use agents to prevent these diseases.

Peroxisome proliferator-activated receptor *γ* (PPAR*γ*) is a nuclear receptor that regulates adipocyte differentiation and metabolism in adipocyte cells. Thiazolidinedione-type compounds (TZDs), which are potent PPAR*γ* ligands, improve insulin sensitivity by increasing concentrations of adiponectin and by decreasing free fatty acid (FFA) and inflammatory factor TNF-*α* levels in diabetic subjects and animal models in vivo and in vitro [21, 22]. It is well established that PPAR*γ* ligands promote insulin sensitivity by promoting adipocyte cell differentiation. Differentiated small adipocyte cells secrete adiponectin and recover insulin sensitivity. Adiponectin promotes the HDL-cholesterol level of blood and ameliorates blood lipid disorders. Furthermore, some dietary compounds show not only improvement of differentiation of adipocytes but also regulation of adipocytokine secretion related to inflammation of adipocytes [23]. Therefore, the compounds which promote adipocyte cell differentiation and which attenuate inflammation in adipocytes are used for the development of antidiabetic resources.

This study examined the recuperative effects of insulin resistance in adipocyte cells by paprika. Promotion of adipocyte cell differentiation and adiponectin secretion effects were investigated using 3T3-L1 mouse preadipocyte cells. Moreover, using the adipocyte inflammation model, a coculture of adipocytes and macrophages and regulation effect of inflammation cytokine were examined. Our data suggest that paprika carotenoids are useful for suppressing the enhanced inflammatory response of adipose tissue.

## 2. Materials and Methods

### 2.1. Chemical Reagents and Cells

Mouse 3T3-L1 preadipocyte cells and RAW264.7 cell were obtained from DS Pharma Biomedical Co. Ltd. (Osaka, Japan). Fetal bovine serum (FBS) was purchased from Biological Industries Ltd. (Kibbutz, Beit, Israel). Dulbecco's modified Eagle's medium (DMEM) was purchased from Nissui Pharmaceutical Co., Ltd. (Tokyo, Japan). Troglitazone was purchased from Funakoshi Co., Ltd. (Tokyo, Japan). PPAR*γ* and *β*-actin antibody were purchased from Santa Cruz Biotechnology Inc. (Santa Cruz, CA, USA). All other chemicals, guaranteed to be of reagent or tissue-culture grade, were from Sigma (MO, USA) or Wako Pure Chemical Industries Ltd. (Osaka, Japan).

### 2.2. Preparation and Purification of Paprika Pigments (PPs)

Paprika carotenoids were purchased from LKT Laboratories Inc. (St. Paul, MN, USA). Paprika carotenoids were saponified to obtain nonacylated form carotenoids with fatty acid. Paprika carotenoids (1 g) were mixed with 10% NaOH (10 mL) and saponified for 12 hr at room temperature. Subsequently, the pigments were extracted with diethyl ether and washed several times with distillated water. They were then evaporated to dryness. Purification was conducted using silica gel column chromatography (Silica Gel 60; Nacalai Tesque Inc., Kyoto, Japan) and eluted with hexane: acetone (7 : 3, v/v). Purified PP did not contain capsaicin which is a major pungent ingredient in red pepper. Then, the contained high levels of capsanthin and capsorubin fraction were used as PP. The capsanthin and capsorubin concentrations of PP were analyzed using high-performance liquid chromatography (HPLC) as described in a previous report [24]. Reversed-phase HPLC was conducted (L-7000; Hitachi Ltd.) with a Develosil ODS-UG-5 (250 × 4.6 mm i.d., 5.0 *μ*m particle size; Nomura Chemical Co., Ltd.) fitted with a guard column (10 × 4.0 mm i.d.) containing the same stationary phase. A mixture of methanol and acetonitrile (7 : 3, v/v) at a flow rate of 1.0 mL min^−1^ was used as mobile phase. Capsanthin was monitored at 450 nm using a UV-Vis detector. The capsanthin concentrations of PP were determined using standard capsanthin (CaroteNature GmbH, Lupsingen, Switzerland) and capsorubin. The standard product of capsorubin was determined using MS analysis (Seisan Kaihatsu Kagaku Kenkyusho, Kyoto, Japan).

### 2.3. Cell Culture

3T3-L1 preadipocyte cells and RAW264.7 macrophage cells were cultured in DMEM with 10% FBS, 100 U mL^−1^ penicillin, and 100 *μ*g mL^−1^ streptomycin at 37°C in a humidified atmosphere of 95% air and 5% CO_2_, respectively. Differentiation of 3T3-L1 preadipocytes was conducted as described in an earlier report [24]. After 3T3-L1 cells reached confluence, they were incubated for an additional 24 hr. Then, adipocyte differentiation of 3T3-L1 preadipocytes was initiated using differentiation medium containing 10 *μ*g mL^−1^ insulin, 0.5 mmol L^−1^ isobutylmethylxanthine, and 0.1 *μ*mol L^−1^  dexamethazone for 48 h. The medium was then replaced with DMEM containing 5 *μ*g mL^−1^ insulin (differentiation medium II) and changed to fresh medium every 48 hr for fresh medium II. PPs were added to differentiation medium II as the ethanol solution. The final concentration of ethanol was adjusted to 0.1% to avoid affecting cell growth. The cytotoxicity of PP on 3T3-L1 cells was determined using WST-1 assay [25].

### 2.4. Measurement of GPDH Activity

Glycerol phosphate dehydrogenase (GPDH, EC 1.1.1.8) activity was measured using a commercial assay kit (Primary Cell Co., Ltd., Sapporo, Japan) according to the manufacturer's instructions [24]. 3T3-L1 cells incubated in differentiation medium II containing paprika carotenoids for 144 hr were washed twice with PBS and dissolved in enzyme extract solution. Then, the cell lysate was homogenized using supersonic waves on ice and centrifuged at 10,000 rpm for 5 min at 4°C. The supernatant was used for the measurement of GPDH activity. The protein contents were measured using a DC protein assay kit (Bio-Rad Laboratories Inc., Tokyo, Japan).

### 2.5. RNA Preparation and Real-Time Quantitative PCR

Total RNA of cultured cells was extracted according to the device (Quick Gene mini 80; Fujifilm Corp., Tokyo, Japan) manufacturer's instructions. Then, total RNA was reverse-transcribed with a High Capacity cDNA Reverse Transcription (Applied Biosystems Japan Ltd., Tokyo, Japan) for cDNA synthesis. PPAR*γ*, adiponectin, resistin, MCP-1, IL-6, and TNF-*α* mRNA expressions were measured using a real-time PCR detection system (Opticon; Bio-Rad Laboratories Inc., CA, USA) and Thunderbird qPCR Mix (Toyobo Co., Ltd., Osaka, Japan). The primer sequences used for RT-PCR were the following: 5′-CAT GGC CTT CCG TGT TCC TA-3′ (forward) and 5′-GCG GCA CGT CAG ATC CA-3′ (reverse) for mouse GAPDH (NM_008084.2); 5′-GCC CAC CAA CTT CGG AAT C-3′ (forward) and 5′-TGC GAG TGG TCT TCC ATC AC-3′ (reverse) for mouse PPAR*γ* (NM_001127330.1); 5′-AAC CCC TGG CAG GAA AGG-3′ (forward) and 5′-TGA ACG CTG AGC GAT ACA CAT-3′ (reverse) for mouse adiponectin (NM_009605.4); 5′-GCT GCT GCC AAG GCT GAT-3′ (forward) and 5′-TCT CCT TCC ACC ATG TAG TTT CC-3′ (reverse) for mouse resistin (NM_022984.3); 5′-CTG AAG CCA GCT CTC TCT TCC T-3′ (forward) and 5′-CAG GCC CAG AAG CAT GAC A-3′ (reverse) for mouse MCP-1 (NM_011333); 5′-CCA CGG CCT TCC CTA CTT C-3′ (forward) and 5′-TTG GGA GTG GTA TCC TCT GTG A-3′ (reverse) for mouse IL-6 (NM_031168.1); 5′-CAC AAG ATG CTG GGA CAG TGA-3′ (forward) and 5′-TCC TTG ATG GTG GTG CAT GA-3′ (reverse) for mouse TNF-*α* (NM_013693.2). Each PCR reaction was normalized to GAPDH.

### 2.6. Western Blot Analysis

3T3-L1 cells were incubated in differentiation medium II containing carotenoid for 144 hr. Cells were lysed with cold RIPA buffer (pH 7.4) containing 20 mmol L^−1^ Tris-HCl, 150 mmol L^−1^ NaCl, 1% NP-40, 0.5% sodium deoxycholate, 0.1% sodium dodecyl sulfate (SDS), 0.1 mg mL^−1^ phenylmethylsulfonyl fluoride, 50 *μ*g mL^−1^ aprotinin, and 1 mmol L^−1^ Na_3_VO_4_. Cell lysates were centrifuged at 12,000 rpm for 20 min at 4°C, and the supernatant (30 *μ*g protein/lane) was separated by 10% SDS-polyvinylidene difluoride membrane. The membrane was incubated with an antibody against PPAR*γ* for 1 hr and then with secondary antibody rabbit IgG-conjugated horseradish peroxidase (Santa Cruz Biotechnology Inc., CA, USA) for 1 hr at room temperature. The membranes were treated with reagents (Chemi-Lumi One L; Nacalai Tesque Inc., Kyoto, Japan) according to the manufacturer's instructions. *β*-Actin was used as the control with the anti-*β*-Actin antibody (Santa Cruz Biotechnology Inc., CA, USA).

### 2.7. Measurement of Adiponectin and Resistin Production

Concentrations of adiponectin and resistin in the culture supernatants were determined using ELISA, conducted using a mouse/rat adiponectin ELISA Kit (Otsuka Pharmaceuticals Co., Ltd., Tokyo, Japan) and Mouse Resistin Elisa Kit (Shibayagi Co., Ltd., Gunma, Japan) in accordance with the manufacturer's instructions.

### 2.8. Coculture of Adipocyte and Macrophage System

3T3-L1 adipocyte cells and RAW264.7 macrophages cells were cocultured in a contact system. 3T3-L1 cells were cultured in differentiation medium I for 48 hr and II for 144 hr. Then, RAW264.7 cells (5 × 10^4^ cells/well) were plated onto 24-well-plate cultured-differentiated 3T3-L1 cells. They were treated with DMEM medium containing PP for 24 hr. Subsequently, cells and culture medium supernatants were collected.

### 2.9. Measurement of Nitric Oxide Release

The amount of nitrite in cell-free culture supernatants measured using Griess Reagents R1 and R2 (Cayman Chemical Co., MI, USA) [26]. Briefly, 100 *μ*L of supernatant was mixed with 50 *μ*L Griess Reagent R1. Immediately after addition of R1, 50 *μ*L Griess Reagent R2 was added. After 10 min, absorbance was measured at 550 nm (iMark; Bio-Rad Laboratories Inc., CA, USA).

### 2.10. Agonistic Activity for PPAR*γ*

Agonistic activity for PPAR*γ* was examined using a nuclear receptor cofactor assay system (EnBio RCAS for PPAR*γ*; COSMO BIO Co., Ltd., Tokyo, Japan). This system is a cell-free assay system using nuclear receptor and cofactor to screen chemicals. The changes of absorbance (450 nm) at PP (50, 100 *μ*g mL^−1^) and troglitazone (10 *μ*mol L^−1^) were measured. 

### 2.11. Statistical Analysis

The results were expressed as mean ± standard deviation (SE). Statistical analyses between multiple groups were determined using ANOVA.

Statistical comparisons were assessed using Dunnett's multiple comparison test. Differences were inferred as significant at *P* < 0.05. Analyses were conducted using software (Stat View-J ver. 5.0; Abacus Concepts Inc., CA, USA).

## 3. Results

### 3.1. Effects of PP on Adipocyte Differentiation of 3T3-L1 Preadipocytes

**Figure 2:**
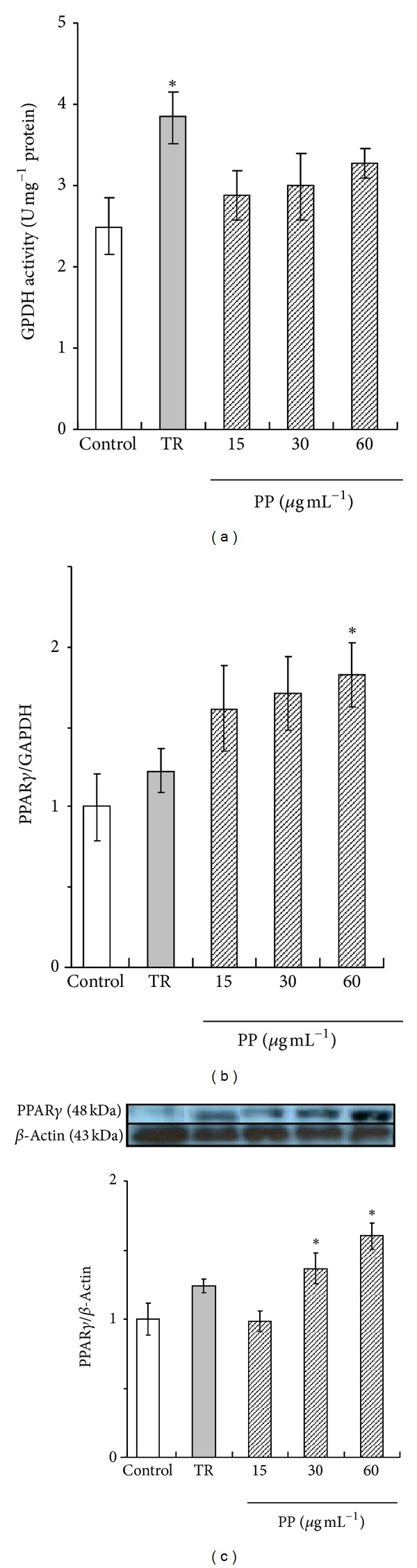
Effects of paprika pigments (PPs) on differentiation of 3T3-L1 adipocyte cells. 3T3-L1 cells were incubated with 10 *μ*mol L^−1^ troglitazone (TR) or 15, 30, or 60 *μ*g mL^−1^ PP for 144 hr. (a) GPDH (glycerol-3-phosphate dehydrogenase) activity. (b) PPAR*γ* mRNA expression. (c) PPAR*γ* protein expression. *P < 0.05 versus control.

First, to clarify the effects of PP on adipocyte metabolites, we examined differentiation of 3T3-L1 preadipocytes in the presence of PP. The main contents of PP, as ascertained using HPLC analysis, were capsanthin (44.3%), capsorubin (12.8%), and analog of capsanthin. The GPDH activity indicates the rate phase of adipocyte differentiation. As presented in Figure 2(a), after treatment with troglitazone (10 *μ*mol L^−1^), a PPAR*γ* ligand, GPDH activity became significantly higher than that of control. Treatment with PP cells tended to be upregulated by 3T3-L1 cell differentiation such as troglitazone.

PP was shown to promote adipocyte differentiation. The next consideration was whether PP would upregulate expression of PPAR*γ*, which is a central determinant of the transcriptional cascade inducing adipocytes. PPAR*γ* mRNA expression was measured using real-time RT-PCR. The expression level treated with 60 *μ*g mL^−1^ PP was significantly higher (*P* < 0.05) than that in control cells (Figure 2(b)). Moreover, PPAR*γ* protein expression treated with 30, 60 *μ*g mL^−1^ paprika carotenoid was promoted significantly more (*P* < 0.05) than in control cells (Figure 2(c)).

### 3.2. Effect of PP on Adipocytokine Production in 3T3-L1 Adipocytes

**Figure 3:**
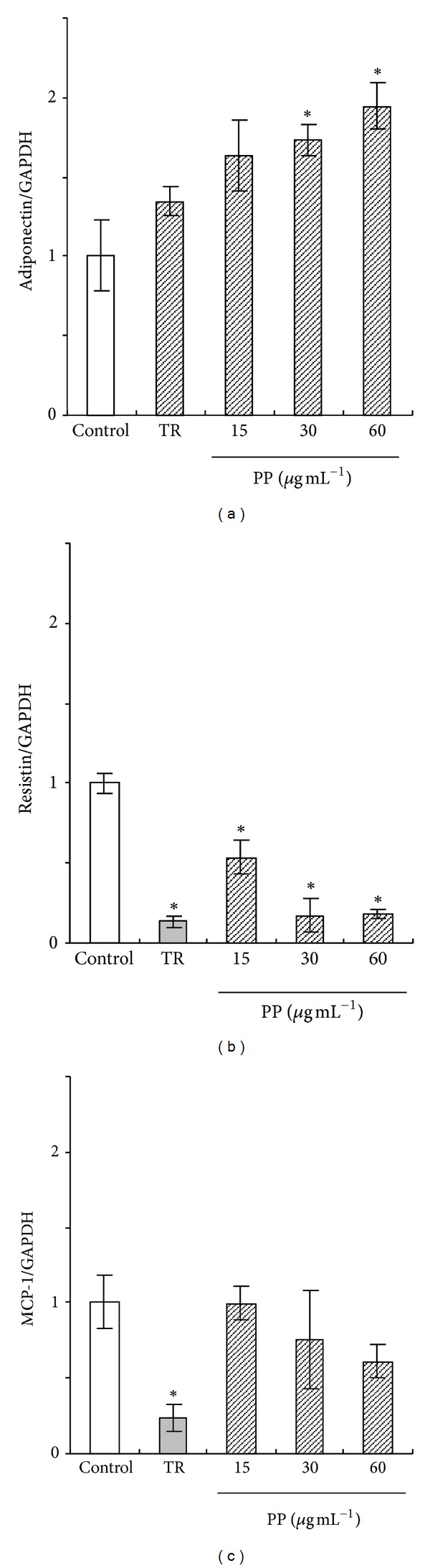
Effects of PP on adiponectin, resistin, and MCP-1 mRNA expression of 3T3-L1 adipocyte cells. 3T3-L1 cells were incubated with 10 *μ*mol L^−1^ troglitazone (TR) or 15, 30, or 60 *μ*g mL^−1^ PP for 144 hr. (a) adiponectin, (b) resistin, and (c) MCP-1. *P < 0.05 versus control.

**Figure 4:**
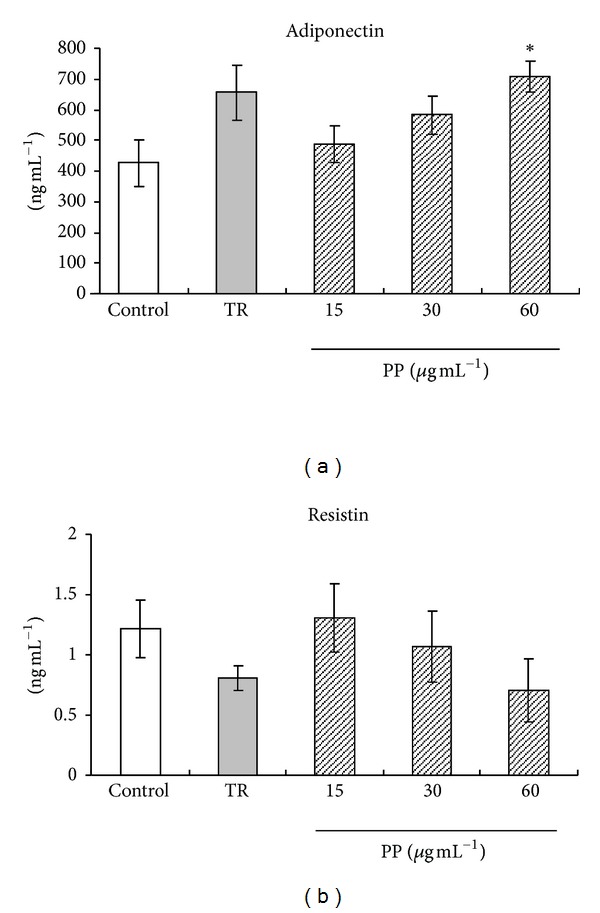
Effects of PP on adiponectin, resistin protein secretion on 3T3-L1 adipocyte cells. 3T3-L1 cells were incubated with 10 *μ*mol L^−1^ troglitazone (TR) or 15, 30, or 60 *μ*g mL^−1^ PP for 144 hr. (a) adiponectin, (b) resistin, and (c) MCP-1. *P < 0.05 versus control.

Adiponectin, resistin, and MCP-1 mRNA expression levels of 3T3-L1 cells treated with PP for 144 hr were measured using real-time RT-PCR. Treated troglitazone cells showed upregulated adiponectin mRNA expression (Figure 3(a)). In contrast, resistin and MCP-1 mRNA levels were downregulated (Figures 3(b) and 3(c)). Adiponectin mRNA expression level was promoted in cells treated dose-dependently with PP (Figure 3(a)). Conversely, resistin mRNA expression level was suppressed significantly (*P* < 0.05) in cells treated with PP (Figure 3(b)). MCP-1 mRNA expression level treated with PP tended to show a low level compared with control cells (Figure 3(c)). Moreover, both adiponectin and resistin secretion from 3T3-L1 adipocyte were examined using ELISA (Figure 4). Adiponectin secretion tended to increase when treated with PP cells. The level was increased significantly (*P* < 0.05) to 150% of the control level at the PP concentration of 60 *μ*g mL^−1^ (Figure 4(a)). In contrast, resistin secretion of the treated PP cells tended to decrease, as did the mRNA expression level (Figure 4(b)).

### 3.3. Effect of PP on the Induction of Inflammatory Changes by Coculture of Adipocytes and Macrophages

**Figure 5:**
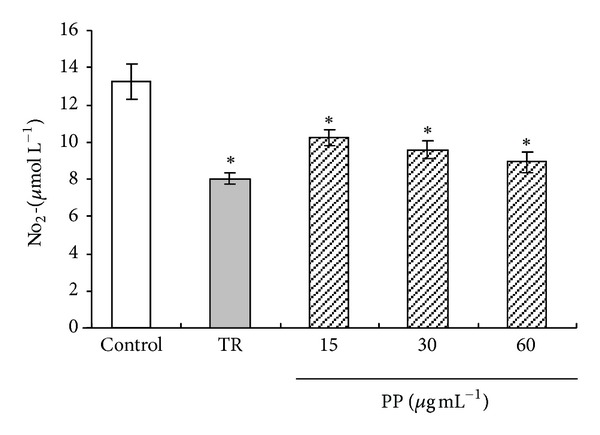
Effects of PP on NO secretion in coculture of 3T3-L1 adipocyte cells and RAW264.7 macrophage cells system. *P < 0.05 versus control.

IL-6, MCP-1, resistin, and TNF-*α* are major adipocytokines that promote inflammatory action in adipocytes. Troglitazone suppressed this cytokine mRNA expression in coculture of 3T3-L1 adipocytes and RAW264.7 macrophage system (Table 1). Treatment with PP cells suppressed mRNA expression compared to that in control cells. Moreover, cells treated with PP showed significantly decreased (*P* < 0.05) NO production dose dependently (Figure 5). These results suggest that PP ameliorates inflammatory changes in adipocyte cells induced by macrophage migration. However, adiponectin mRNA expression was not increased significantly, as it was in cultured 3T3-L1 adipocytes cells alone.

### 3.4. Agonistic Activity of PP for PPAR*γ*

PPAR*γ* agonistic activity was examined using a nuclear receptor cofactor assay system. The absorbance of nontreated well (control) was estimated as 100%; change of absorbance percentage was calculated. Absorbance percentage of control was 100.0 ± 11.4%, treated with troglitazone (10 *μ*mol L^−1^) was 306.6 ± 14.9%. Treated with PP (50, 100 *μ*g mL^−1^) were significantly higher (*P* < 0.05) than that of control (PP 50 *μ*g mL^−1^; 155.4 ± 2.6%, PP 100 *μ*g mL^−1^; 156.3 ± 6.3%). 

## 4. Discussion

In this study, we demonstrated the effect of PP, which contains capsanthin and capsorubin, on adipocyte cells. Treatment with PP 3T3-L1 preadipocyte cells increased the activity of GPDH, which is an enzyme related to fat accumulation in adipocyte cells. Moreover, PP enhanced the mRNA expression and protein level of PPAR*γ* in 3T3-L1 cells. PPAR*γ* plays an important role in the early stages of 3T3-L1 cell differentiation because it is a nuclear transcription factor that regulates adipogenic gene expression [27–29]. These results suggest that PP has the activity of promoting adipocyte cell differentiation. Reportedly, an increase in the number of small adipocytes can improve insulin resistance [30]. Thiazolidinediones show antidiabetic effects by promoting adipocyte differentiation through the increased number of small adipocytes. Small adipocyte cells show the effect of recovering insulin resistance, which is attributed to adiponectin secretion [31]. In this study, treated PP cells exhibited increased adiponectin secretion and decreased resistin secretion, which is related to impaired glucose homeostasis. This effect was related to agonist activity of PP for PPAR*γ*. Consequently, these results suggest that PP improves glucose tolerance by increasing the number of small adipocytes.

Recent reports have described that obesity is an inflammatory disease that causes insulin resistance in adipose tissues, skeletal muscle, and the liver [32, 33]. Obese adipose tissues are characterized by enhanced infiltration of macrophages. A paracrine loop involving adipocyte is believed to derive free fatty acids [34]. IL-6, MCP-1, resistin, and TNF-*α* are associated strongly with obesity-induced inflammation and obesity-related pathologies. In this study, PP suppressed these gene mRNA expressions and NO secretion in coculture of adipocytes and macrophages. The effect is similar to that of troglitazone, which is a PPAR*γ* ligand such as TZDs. These results indicate that PP treatment attenuates inflammation in obesity-induced inflammatory adipocyte cells.

In this study, the effects of capsanthin and capsorubin, which are purified from paprika, were determined. Paprika contains mainly capsanthin, but it also has capsorubin, capsanthin 3,6-epoxide, and *β*-carotene. Reportedly, *β*-carotene accumulation in 3T3-L1 adipocytes suppresses gene expression related to insulin sensitivity [35]. Furthermore, these carotenoids show antioxidative activity. Capsorubin isolated from paprika is a more effective antioxidant than *β*-carotene [36]. Reportedly, antioxidant (*N*-acetylcysteine) prevents obesity-induced metabolic changes in 3T3-L1 adipocytes [37]. Therefore, the antioxidative activity of carotenoids might be related to the attenuation of inflammation in obese adipocytes. Additionally, PP inhibited NO production in adipocyte cells induced by macrophage migration (Figure 5). Capsanthin and capsorubin in paprika exert suppressive effects of NO production in LPS-stimulated RAW264.7 cells, which are associated with inflammation [13]. NO produced from activated macrophages is associated with acute and chronic inflammation. Toll-like receptor 4 (TLR4) is regarded as a necessary cell surface receptor for the recognition of LPS macrophages. Free fatty acids released from adipocytes by the lipolysis of triglycerides exert proinflammatory effects on macrophages through the activation of TLR4 [38]. TLR4 regulates several transcription factors encoding inflammatory mediators. NF-*κ*B is an important transcription factor adjusting proinflammatory mediators' production in activated macrophages. It has been shown that PPAR*γ* ligands inhibit monocyte/macrophage chemotaxis and activation. PPAR*γ* ligands have been shown to suppress intranuclear NF-*κ*B pathway, leading to the suppression of the release of proinflammatory mediators [39]. In present study, PP showed PPAR*γ* agonistic activity. Therefore, carotenoids in paprika probably suppressed activation of NF-*κ*B, regulated TLR4 signaling of macrophages, and recovered chronic inflammation in adipocyte cells induced by metabolic disorder.

## 5. Conclusions

PP including capsanthin and capsorubin recover insulin resistance by promoting the differentiation and adiponectin secretion in 3T3-L1 adipocyte cells. In addition, PP attenuates inflammatory changes in the interaction between adipocytes and macrophages. Therefore, our results suggest that consumption of carotenoids in paprika contributes to the partial prevention and improvement of obesity-related insulin resistance.

## Figures and Tables

**Table 1:** Effects of PP on adipocytokine and chemokine mRNA expression in a co-culture of 3T3-L1 adipocyte cells and a RAW264.7 macrophage cells system. RAW264.7 cells were plated onto cultured differentiated 3T3-L1 adipocyte cells. They were treated with medium containing 10 *μ*mol L^−1^ troglitazone (TR) or 15, 30, and 60 *μ*g mL^−1 ^PP for 24 hr.

	Control	TR (10 *μ*mol L^−1^)	PP (15 *μ*g mL^−1^)	PP (30 *μ*g mL^−1^)	PP (60 *μ*g mL^−1^)
IL-6/GAPDH	1.00 ± 0.24	0.78 ± 0.05	0.41 ± 0.02*	0.38 ± 0.08*	0.24 ± 0.02*
MCP-1/GAPDH	1.00 ± 0.17	0.43 ± 0.14*	1.06 ± 0.29	0.72 ± 0.07*	0.51 ± 0.03*
Resistin/GAPDH	1.00 ± 0.66	0.21 ± 0.16*	0.25 ± 0.18*	0.14 ± 0.11*	0.08 ± 0.03*
TNF-*α*/GAPDH	1.00 ± 0.09	0.56 ± 0.08*	0.86 ± 0.07	0.75 ± 0.05	0.65 ± 0.05*
Adiponectin/GAPDH	1.00 ± 0.30	1.12 ± 0.18	1.33 ± 0.20	1.21 ± 0.23	0.98 ± 0.27

*P < 0.05 versus control.
